# Treatment of high cervical arteriovenous fistulas in the craniocervical junction region

**DOI:** 10.3389/fneur.2023.1164548

**Published:** 2023-06-27

**Authors:** Han Su, Jinlu Yu

**Affiliations:** Department of Neurosurgery, First Hospital of Jilin University, Changchun, China

**Keywords:** craniocervical junction, high cervical cord, arteriovenous fistula, treatment, review

## Abstract

The craniocervical junction (CCJ) is a complex region. Rarely, arteriovenous fistulas (AVFs) can occur in the CCJ region. Currently, it is accepted that CCJ AVFs should only refer to AVFs at the C1-C2 levels. It is reasonable to assume that high cervical CCJ AVFs are being referred to when discussing CCJ AVFs. High cervical CCJ AVFs can be divided into the following four types: dural AVF, radicular AVF, epidural AVF and perimedullary AVF. Until now, it was difficult to understand high cervical CCJ AVFs and provide a proper treatment for them. Therefore, an updated review of high cervical CCJ AVFs is necessary. In this review, the following issues are discussed: the definition of high cervical CCJ AVFs, vessel anatomy of the CCJ region, angioarchitecture of high cervical CCJ AVFs, treatment options, prognoses and complications. Based on the review and our experience, we found that the four types of high cervical CCJ AVFs share similar clinical and imaging characteristics. Patients may present with intracranial hemorrhage or congestive myelopathy. Treatment, including open surgery and endovascular treatment (EVT), can be used for symptomatic AVFs. Most high cervical CCJ AVFs can be effectively treated with open surgery. EVT remains challenging due to a high rate of incomplete obliteration and complications, and it can only be performed in superselective AVFs with simple angioarchitecture. Appropriate treatment can lead to a good prognosis.

## 1. Introduction

The craniocervical junction (CCJ) is a complex bony region (1). Arteriovenous fistulas (AVFs) are arteriovenous shunting diseases without a nidus that rarely occur in the CCJ region, accounting for 1–2% of all cerebrospinal AVFs (2, 3). CCJ AVFs are uniquely complex due to their inherent angioarchitecture (4–6). CCJ AVFs may present with intracranial hemorrhage due to AVF rupture or edema of the brainstem and upper cervical cord due to venous hypertension (7, 8). For these CCJ AVFs, prompt treatment is needed, mainly including open surgery or endovascular treatment (EVT) (3).

EVT can only be used in certain CCJ AVFs because the feeding arteries of most CCJ AVFs are too small and tortuous, and EVT will result in incomplete obliteration, high recurrence and a risk of complications (9). Currently, open surgery still serves as the first-line treatment for CCJ AVFs. However, open surgery is challenging because of the deep-seated location and complex angioarchitecture of CCJ AVFs (10). Until now, it was difficult to understand the angioarchitecture and provide a proper treatment for CCJ AVFs. Therefore, an updated review of CCJ AVFs is necessary.

## 2. Definition of high cervical CCJ AVFs

**Figure 1:**
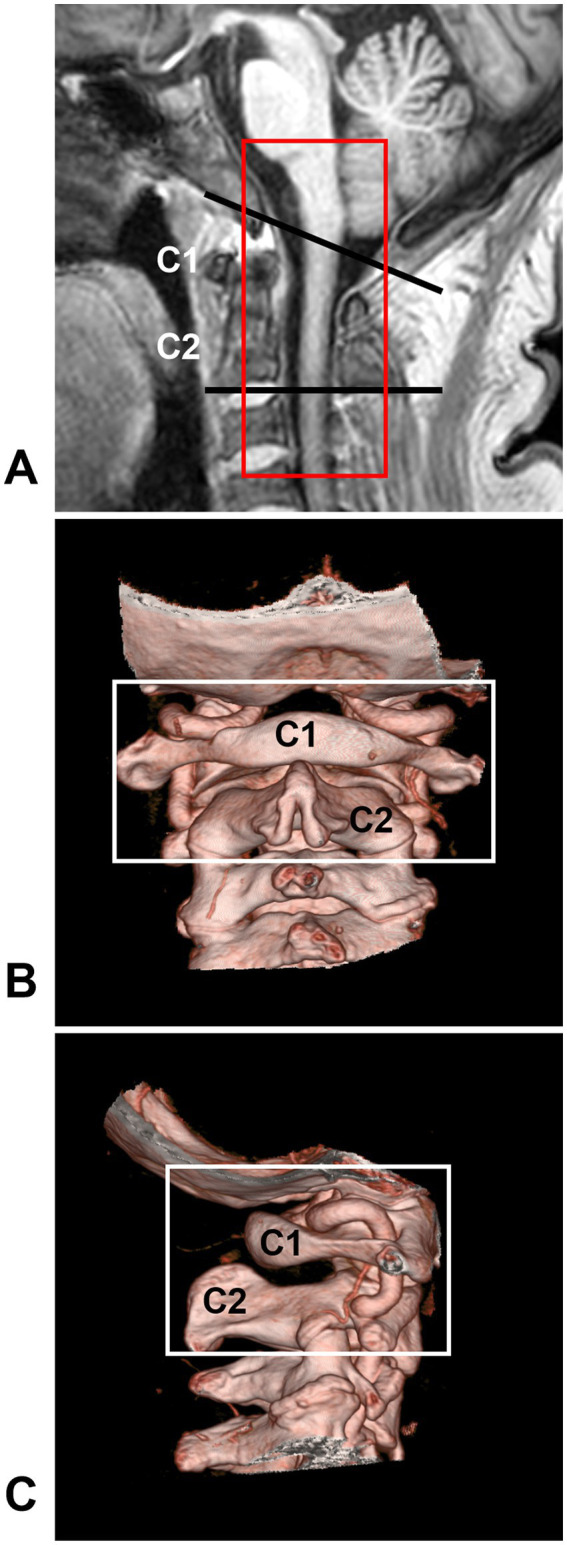
CCJ region definition. **(A)** MRI showing that the CCJ region was located between two black lines and formed by the occipital bone and C1-2 vertebrae. The red frame indicates the area often involved in CCJ AVF. **(B,C)** CTA showing the bony CCJ region (frames). AVF, arteriovenous fistula; C1-2, first and second cervical vertebrae; CCJ, craniocervical junction; CTA, computed tomography angiography; MRI, magnetic resonance imaging.

The CCJ region is located between the skull base and the upper cervical spine and is formed by the occipital bone and the first two cervical vertebrae (C1 or atlas and C2 or axis), which span both the lower brainstem and upper cervical cord (Figure 1) (2, 11, 12). The junction of the lower brainstem and upper cervical cord is situated at the level of the most rostral rootlets of the C1 nerve (13, 14). AVFs that occur in the CCJ region are called CCJ AVFs.

Broadly speaking, CCJ AVFs should contain foramen magnum (FM) DAVFs (15–17). FM DAVFs are located at the anterior condylar confluence, anterior condylar vein (hypoglossal canal), posterior condylar vein (posterior condylar canal), marginal sinus or jugular foramen (18–22). They share similar clinical characteristics and angioarchitectures, and their feeding arteries are derived from the ascending pharyngeal artery (AphA), occipital artery (OA), and posterior meningeal artery of the vertebral artery (VA); their veinous drainage flows into the adjacent sinuses or condylar vein system (17, 20, 23–31).

CCJ AVFs at the C1-C2 levels are different from FM DAVFs, although they include the same elements of FM DAVFs and share a similar drainage pathway (32). Angioarchitectures of CCJ AVFs at the C1-C2 levels are more similar to spinal DAVFs in fistula structure and feeding arteries (4, 30). Therefore, according to some authors’ suggestions, CCJ AVFs should only refer to AVFs at the C1-C2 levels, and FM DAVFs or spinal AVFs at C3 and lower levels should not be included as CCJ AVFs (33–37). In our opinion, we consider the term ‘high cervical CCJ AVFs’ to be more appropriate when discussing CCJ AVFs.

## 3. Vessel anatomy of the high cervical CCJ region

### 3.1. Arterial branches

**Figure 2:**
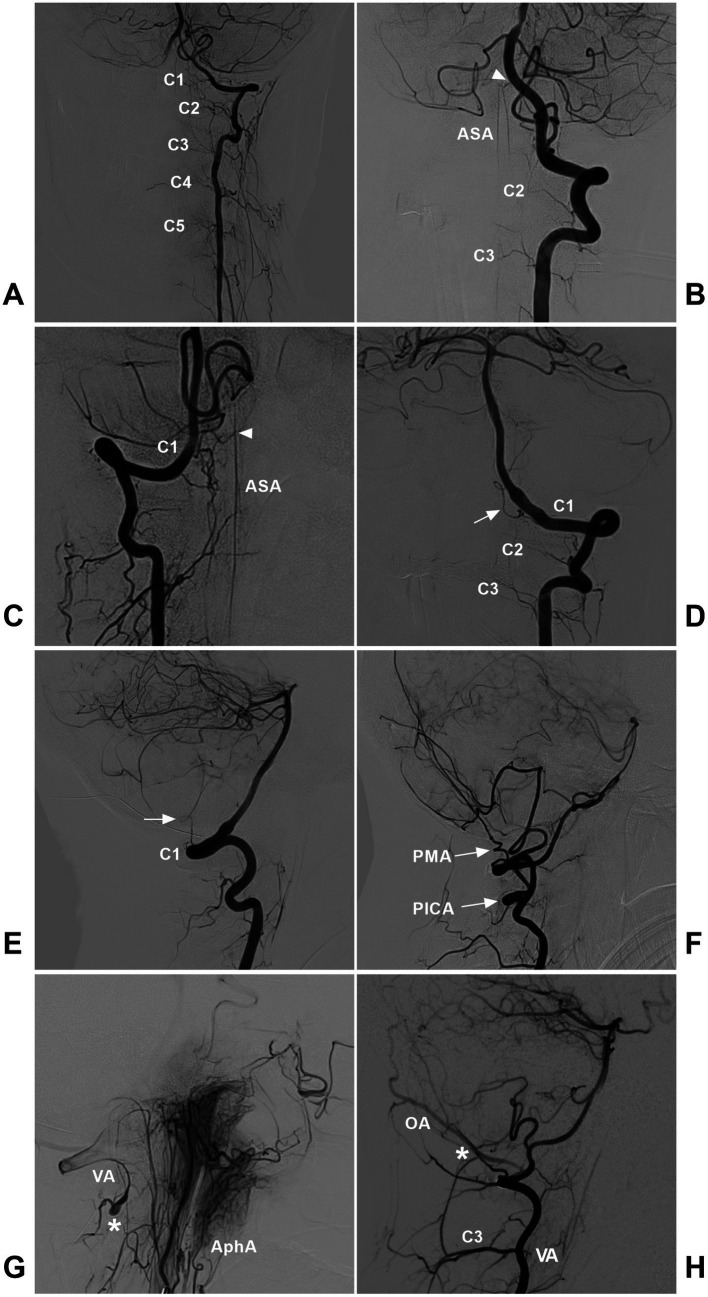
Arteries involved in the high cervical CCJ region. **(A)** DSA showing the radicular arteries from the VA at the C1-C5 levels. **(B)** DSA showing the ASA (arrowhead) from VA termination. The ASA had double trunks, and the radicular arteries of the C2-C3 levels could be seen. **(C)** DSA showing the ASA (arrowhead) originating from the C1 radicular artery. **(D,E)** DSA of anterior posterior **(D)** and lateral **(E)** views showing the C1 radicular artery (arrow). **(F)** DSA showing the extradural low PICA (PICA with arrow) and the PMA (PMA with arrow) from the VA. **(G)** DSA showing the anastomosis (asterisk) between the AphA and VA. **(H)** DSA showing the anastomosis (asterisk) between the OA and muscular branch of the VA. AphA, ascending pharyngeal artery; ASA, anterior spinal artery; C1-C5, first-fifth cervical vertebrae; CCJ, craniocervical junction; DSA, digital subtracted angiography; OA, occipital artery; PICA, posterior inferior cerebellar artery; PMA, posterior meningeal artery; VA, vertebral artery.

The arteries involved in the high cervical CCJ region mainly originate from the suboccipital V3 segment of the VA, including the radicular (or radiculomeningeal, radiculopial or radiculomedullary) artery, anterior and posterior spinal arteries (ASA and PSA), posterior inferior cerebellar artery (PICA), anterior and posterior meningeal arteries, and some muscular branches (Figures 2A–F) (38–40). Additional contributors include the meningeal branches of AphA and OA (Figures 2G,H) (35, 40–42).

The high cervical cord has little radicular inflow, and the radicular arteries are often thin or invisible on digital subtracted angiography (DSA) (Figure 2A) (43, 44). The C1 nerve root is adherent to the VA inferior surface, where the VA gives rise to the C1 radicular artery (Figures 2D,E) (13, 45). The C2 radiculomuscular artery is constant and arises from the VA under the C1 transverse foramen and gives rise to the radicular artery (13, 38, 46). Occasionally, the C3 radicular artery may supply the CCJ region (35). In addition, above the C1 posterior arch, a muscular branch consistently arises from the VA (38). The ASA formed by the union of the paired anterior ventral spinal arteries from intradural VAs or radiculomedullary arteries descends to the CCJ region (Figures 2B,C) (47). The PSAs may arise from the posteromedial surface of the VAs, just outside the dura mater, but they may also be derived from the PICA (48).

### 3.2. Venous drainage

**Figure 3:**
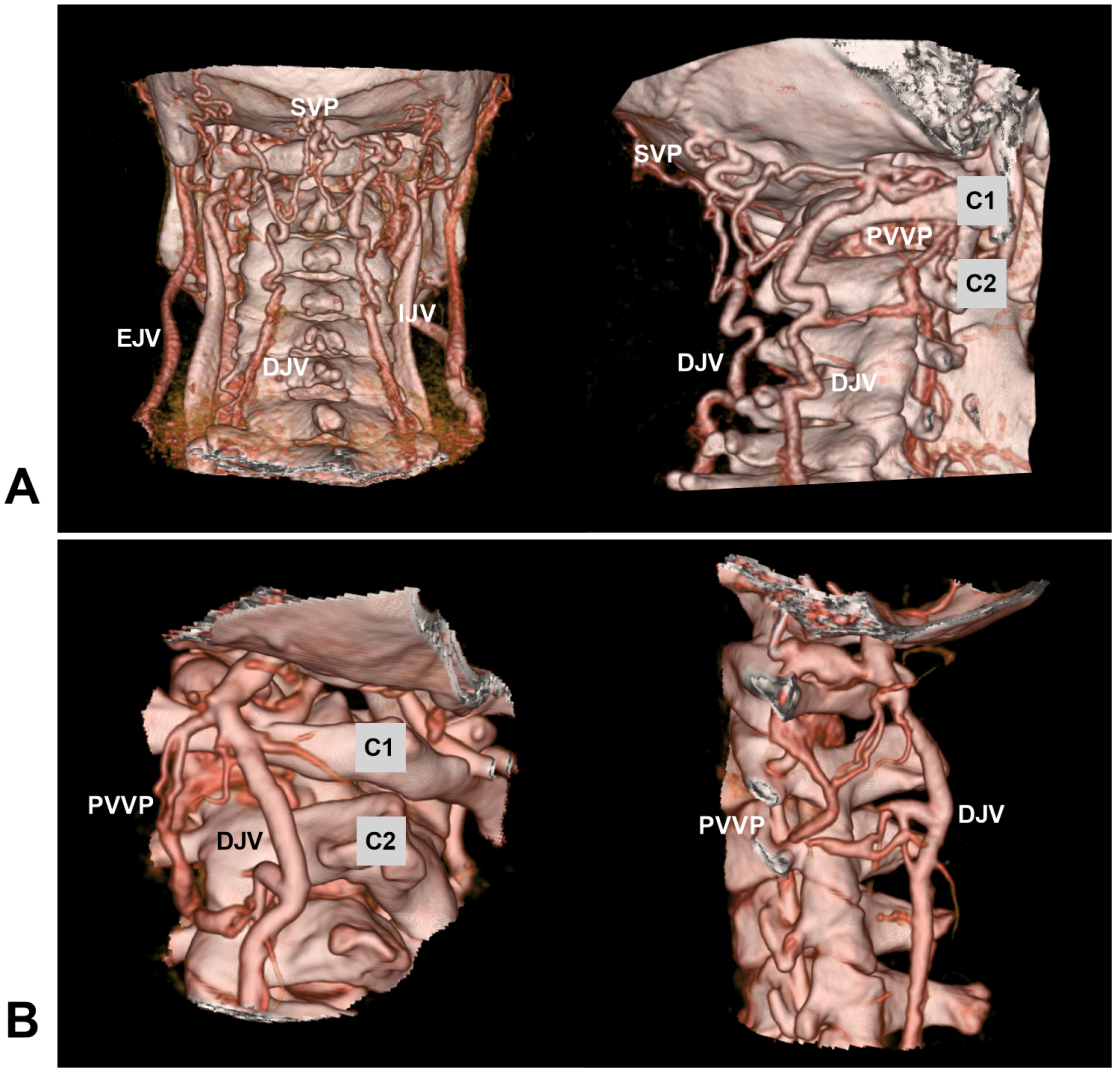
Venous plexuses in the CCJ region. **(A)** Posterior anterior view (left panel) and oblique view (right panel) CTA showing the anastomosed SVP, PVVP, and DJV. **(B)** Oblique view (left panel) and lateral view (right panel) CTA showing the anastomosed PVVP and DJV. C1, C2, first and second cervical vertebrae; CCJ, craniocervical junction; CTA, computed tomography angiography; DJV, deep jugular vein; EJV, external jugular vein; IJV, internal jugular vein; PVVP, paravertebral venous plexus; SVP, suboccipital venous plexus.

Venous drainage in the high cervical CCJ region is abundant, complex and varied, including the extradural and intradural drainage systems (13, 14, 49, 50). The extradural drainage system includes the anastomosed paravertebral and epidural venous plexuses (38, 51). The paravertebral venous plexus communicates with the deep jugular vein, sigmoid sinus and suboccipital venous plexus (Figure 3) (13, 40, 52). The intradural drainage system includes the veins of the lower brainstem and upper cervical cord and anterior/posterior median/lateral spinal veins anastomosed with the veins of the medulla oblongata (13, 14, 34). In addition, several bridging veins collect drainage from the lower brainstem and upper cervical cord into adjacent sinuses or the venous plexus via the radicular vein along nerve rootlets (13, 34).

## 4. Angioarchitecture of high cervical CCJ AVFs

### 4.1. Classification and angioarchitecture

**Figure 4:**
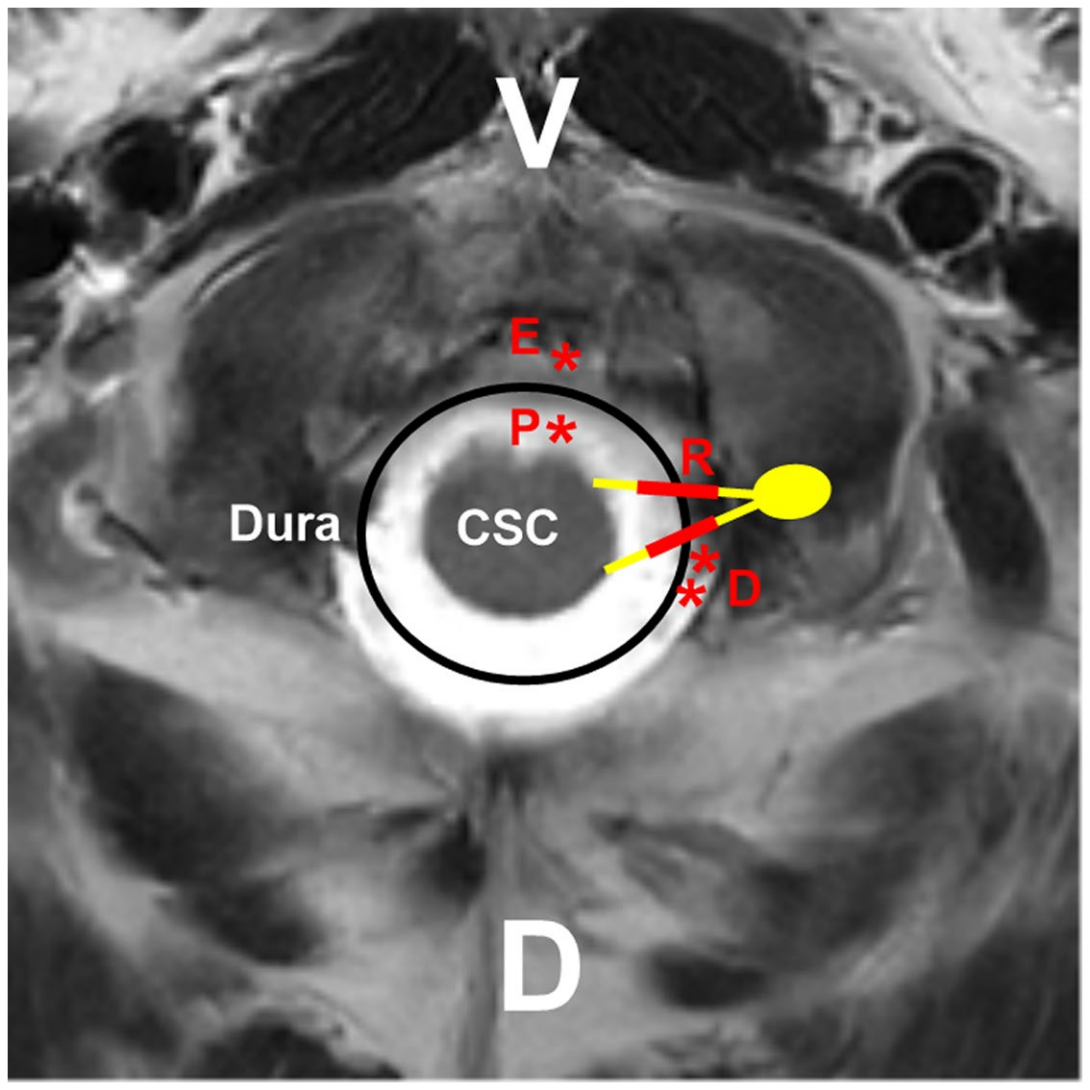
Locations of different high cervical CCJ AVFs. On the hybrid mode pattern of magnetic resonance imaging, the locations of different high cervical CCJ AVFs are shown. PAVF (red P with asterisk) located at the ventral spinal cord surface, RAVF (red R with thick line segments) located at C1-C2 nerves, DAVF (red D with double asterisks) located at the dorsal dura, EAVF (red E with asterisk) in ventral epidural space. AVF, arteriovenous fistula; C1-C2, first and second cervical vertebrae; CCJ, craniocervical junction; CSC, cervical spinal cord; D, dorsal; DAVF, dural AVF; EAVF, epidural AVF; PAVF, perimedullary AVF; RAVF, radicular AVF; V, ventral.

**Figure 5:**
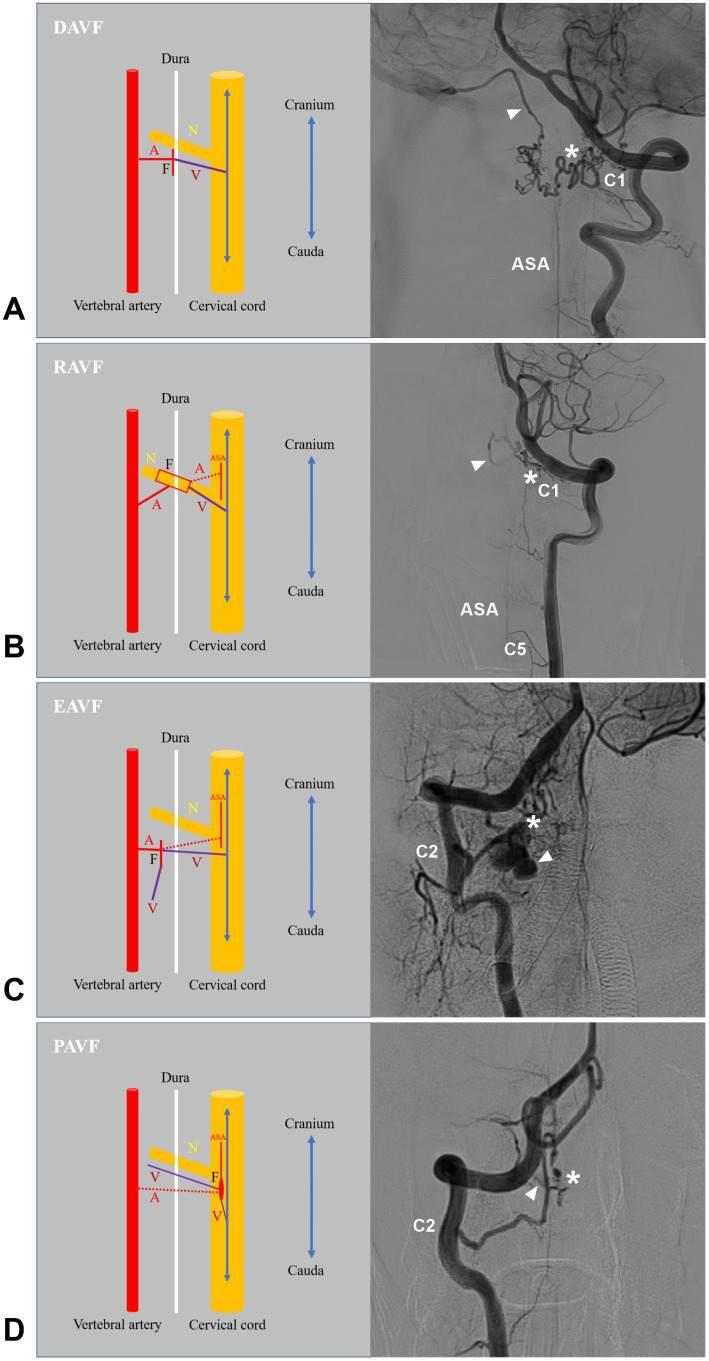
Types of high cervical CCJ AVFs. **(A)** Left panel showing the structure of DAVF in drawing, a radiculomeningeal artery and a radicular vein connected directly on dural sleeve of nerve root; Right panel showing the angioarchitecture of DAVF, the feeding artery was the C1 radicular artery, the asterisk indicated fistula point, and the arrowhead indicate the drainage into intracranial vein to sigmoid sinus. **(B)** Left panel showing the structure of the RAVF in drawing, a radicular (radiculomeningeal) artery and a radicular vein connected directly on the spinal nerve root. The ASA branch can be involved as the feeder (dotted line); right panel showing the angioarchitecture of the RAVF. The feeding artery was the C1 radicular artery, the asterisk indicates the fistula point, and the arrowhead indicates drainage into the intracranial vein. **(C)** Left panel showing the structure of the EAVF in the drawing. There is a direct arteriovenous shunt between the radicular and/or meningeal arteries and the epidural venous plexus. The ASA branch can be involved as the feeder (dotted line); right panel showing the angioarchitecture of the EAVF. The feeding artery is the C2 radicular artery, the asterisk indicates the fistula point, and the arrowhead indicates the drainage. **(D)** Left panel showing the structure of the PAVF in the drawing. The PAVF is a direct arteriovenous communication without an intervening nidus on the surface of the spinal cord; right panel showing the angioarchitecture of the PAVF. The feeding artery was the C2 radiculomedullary artery, the asterisk indicates the fistula point, and the arrowhead indicates the drainage. A, feeding artery; ASA, anterior spinal artery; AVF, arteriovenous fistula; C1, C2, and C5, first, second and fifth cervical vertebrae; CCJ, craniocervical junction; DAVF, dural AVF; EAVF, epidural AVF; F, fistula point; N, nerve; PAVF, perimedullary AVF; RAVF, radicular AVF; V, draining vein.

High cervical CCJ AVFs can be located on the inner or outer surface of the dura mater, on the spinal nerves, or on the spinal cord surface and can be divided into the following four types: DAVFs, radicular AVFs (RAVFs), epidural AVFs (EAVFs), and perimedullary AVFs (PAVFs) (Figures 4, 5), in which DAVFs and RAVFs tend to occur at the C1 level and EAVFs and PAVFs tend to occur at the C2 level (10, 33, 53, 54).

High cervical CCJ AVFs may be single or multiple AVFs, and less than 10% of AVFs are multiple AVFs (4, 7, 55). Multiple AVFs may be homogeneous, such as bilateral DAVFs, bilateral RAVFs or bilateral EAVFs (4, 53, 55–57). More often, multiple AVFs are heterogeneous. Different types of AVFs may coexist due to venous hypertension and thrombosis, such as the combinations of DAVF and PAVF, RAVF and PAVF, DAVF and EAVF and PAVF, and DAVF and RAVF and PAVF (2–4, 35, 53, 58–60).

**Figure 6:**
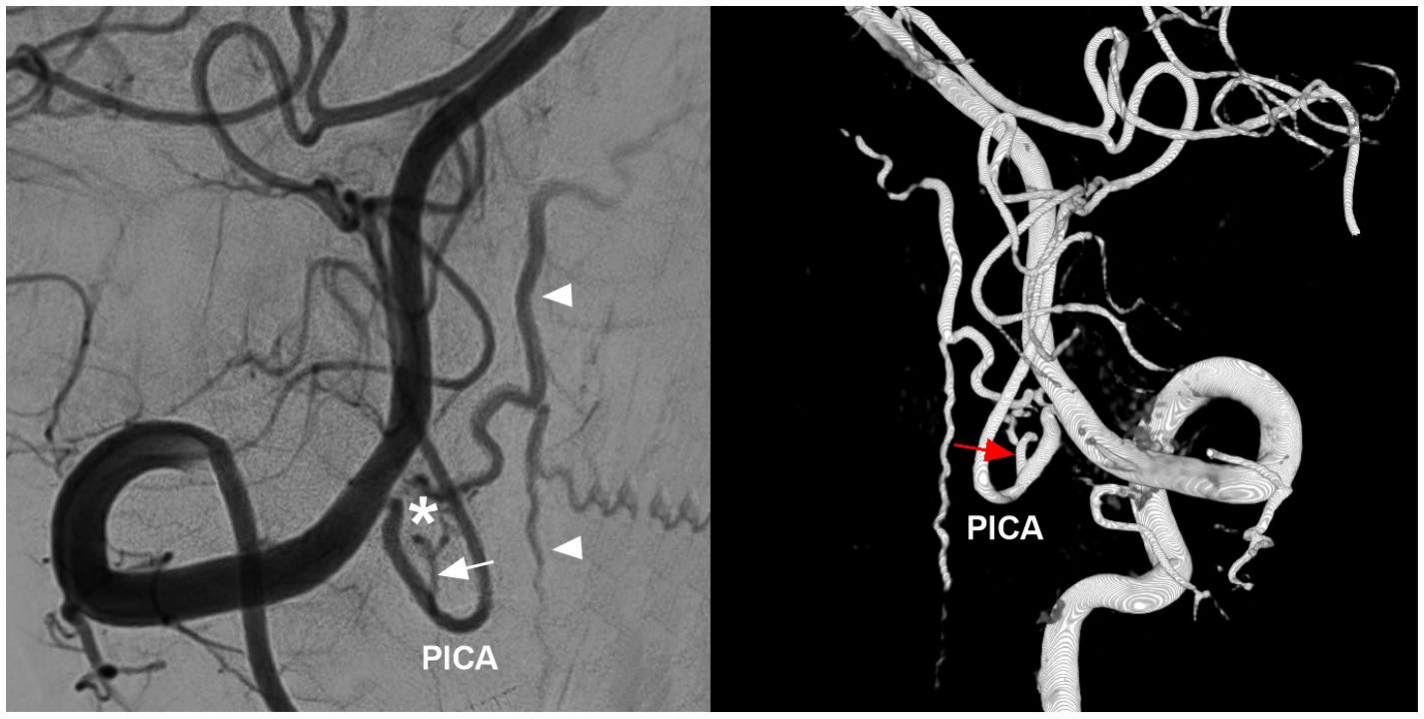
PICA involvement in the feeding artery of high cervical CCJ RAVF. DSA (left panel) and three-dimensional reconstructive DSA (right panel) showing a high cervical CCJ RAVF (asterisk in left panel) fed by the branch of the PICA (arrows); arrowheads in left panel indicate upward and downward draining veins. AVF, arteriovenous fistula; CCJ, craniocervical junction; DSA, digital subtracted angiography; PICA, posterior inferior cerebellar artery; RAVF, radicular AVF.

High cervical CCJ AVFs present with multiple feeding arteries connecting to the same draining vein (5). They are mainly fed by the radicular (radiculomeningeal, radiculopial or radiculomedullary) arteries of the VA (Figure 5) and rarely fed by the branch of the low-positioned PICA (Figure 6) (3, 36). Feeding arteries of high cervical CCJ AVFs may be bilateral or multiple segmental (7, 33, 35). Spinal pial arteries from the ASA and PSA (or posterior lateral spinal artery) can be involved in more than half of CCJ AVFs (33). Sometimes, meningeal branches of the AphA and OA can be involved in the feeders for CCJ DAVFs (7, 33, 61). Due to hemodynamic stress, flow-related aneurysms can occur on the feeding artery (34, 53, 59, 62). The rate is not low; in a report by Song et al. (7) aneurysmal structures occurred in 26.2% of CCJ AVFs.

**Figure 7:**
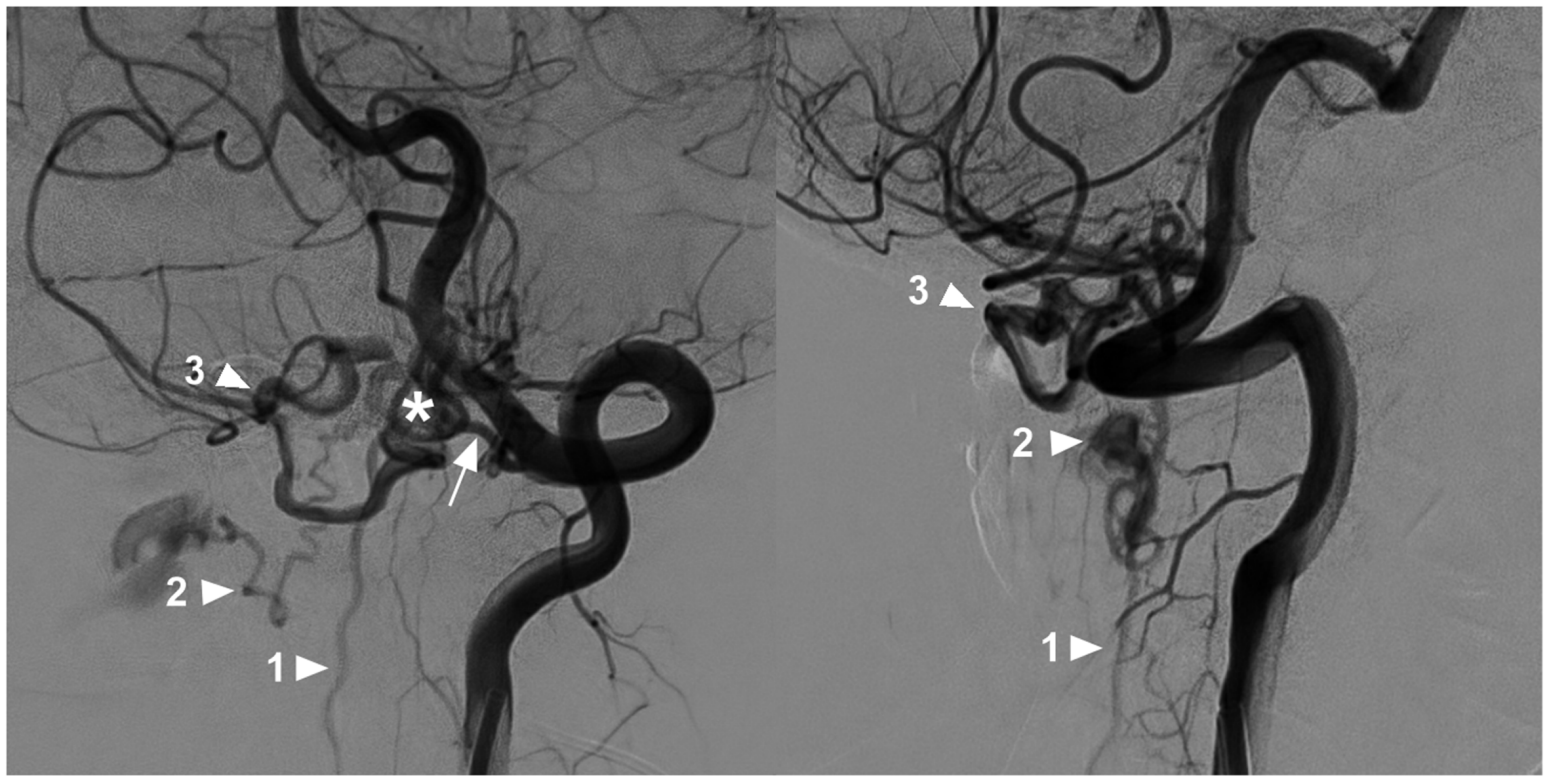
Multiple draining pathways in a high cervical CCJ AVF. Oblique-view DSA of the VA showing a CCJ AVF (asterisk in left panel) supplied by the C1 radicular artery (arrow in left panel); the drainage of the AVF had three pathways: downward into the perimedullary vein (arrowhead with number 1), lateral into the epidural venous plexus (arrowhead with number 2), and upward into the intracranial vein (arrowhead with number 3). AVF, arteriovenous fistula; C1, first cervical vertebrae; CCJ, craniocervical junction; DSA, digital subtracted angiography; VA, vertebral artery.

Most CCJ AVFs had intradural venous drainage, which was ventral to the medulla oblongata or spinal cord (9). Normally, venous drainage of the upper cervical cord flows downward to reach the outlet along the adjacent nerve root by bridging and radicular veins into the lateral epidural venous plexus. When CCJ AVFs occur, due to high hemodynamic pressure, draining veins may extend downward very far, even as far as the thoracic or sacral levels, to reach the outlet (63). High cervical CCJ AVFs also had upward intradural drainage. Even the draining veins can reflux upward into the intracranial venous system due to rich anastomosis (3, 4, 10, 36, 44, 55, 58, 61, 64). Due to the overload of the draining vein, drainage may involve multiple veins or multiple pathways (Figure 7) (33).

**Figure 8:**
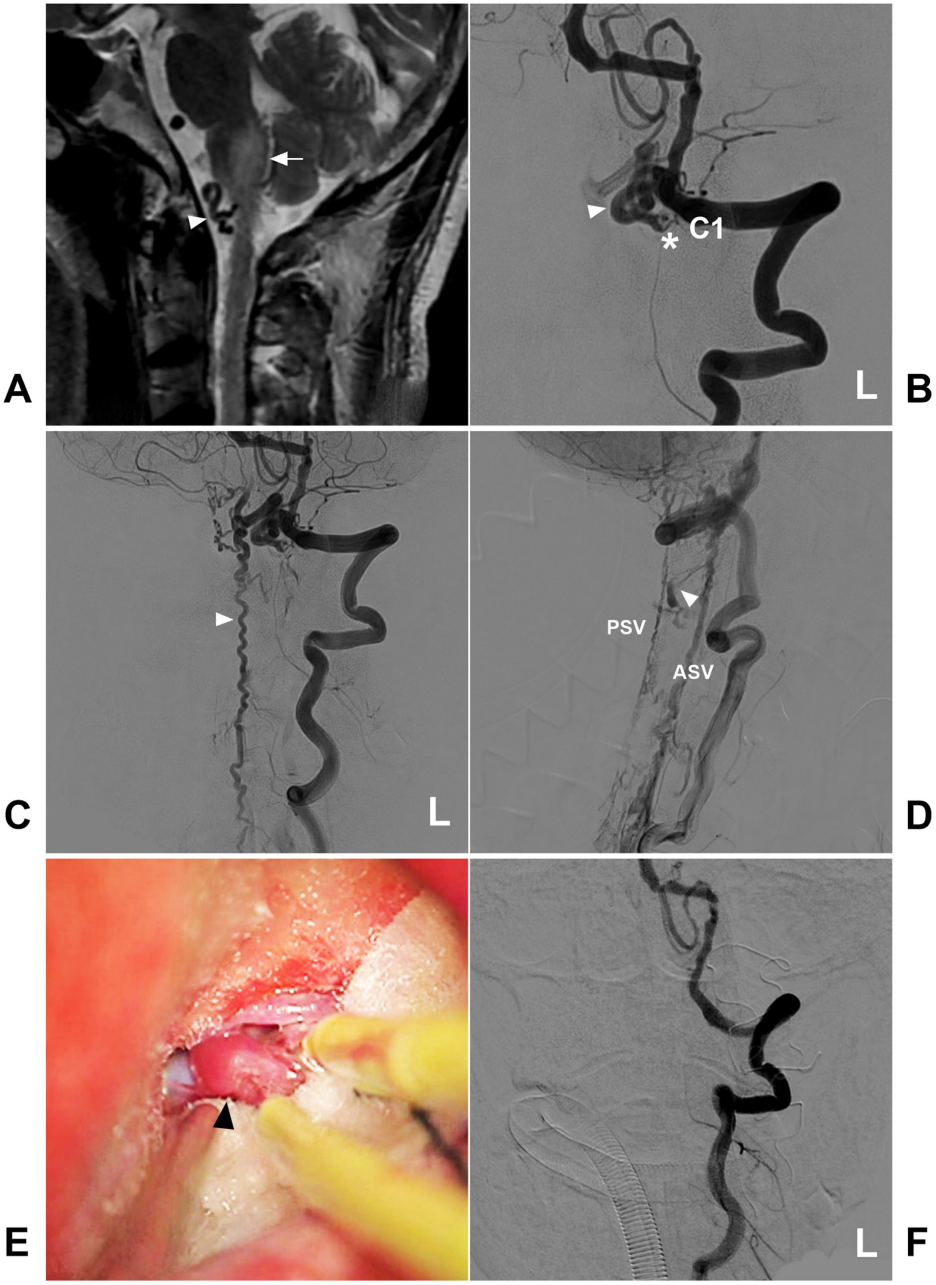
Open surgery in a high cervical CCJ DAVF with downward drainage. **(A)** MRI showing edema (arrow) of the medulla oblongata and abnormal vessels (arrowhead) in the front of the brainstem. **(B)** Arterial phase DSA of the left VA showing a CCJ DAVF (asterisk) fed by the C1 radicular artery. The drainage went into the intracranial vein (arrowhead). **(C)** Late arterial phase DSA of the anterior posterior view showing downward drainage into the perimedullary vein (arrowhead). **(D)** Late arterial phase DSA of the lateral view showing downward drainage, including downward ASV and PSV, and lateral drainage by the bridging and radicular veins (arrowhead). **(E)** Intraoperative image showing the thick arterialized draining vein (arrowhead). **(F)** Postoperative DSA showing that the DAVF was obliterated after the draining vein was coagulated and cut. ASV, anterior spinal vein; C1, first cervical vertebra; CCJ, craniocervical junction; DAVF, dural arteriovenous fistula; DSA, digital subtraction angiography; L, left; MRI, magnetic resonance imaging; PSV, posterior spinal vein; VA, vertebral artery.

**Figure 9:**
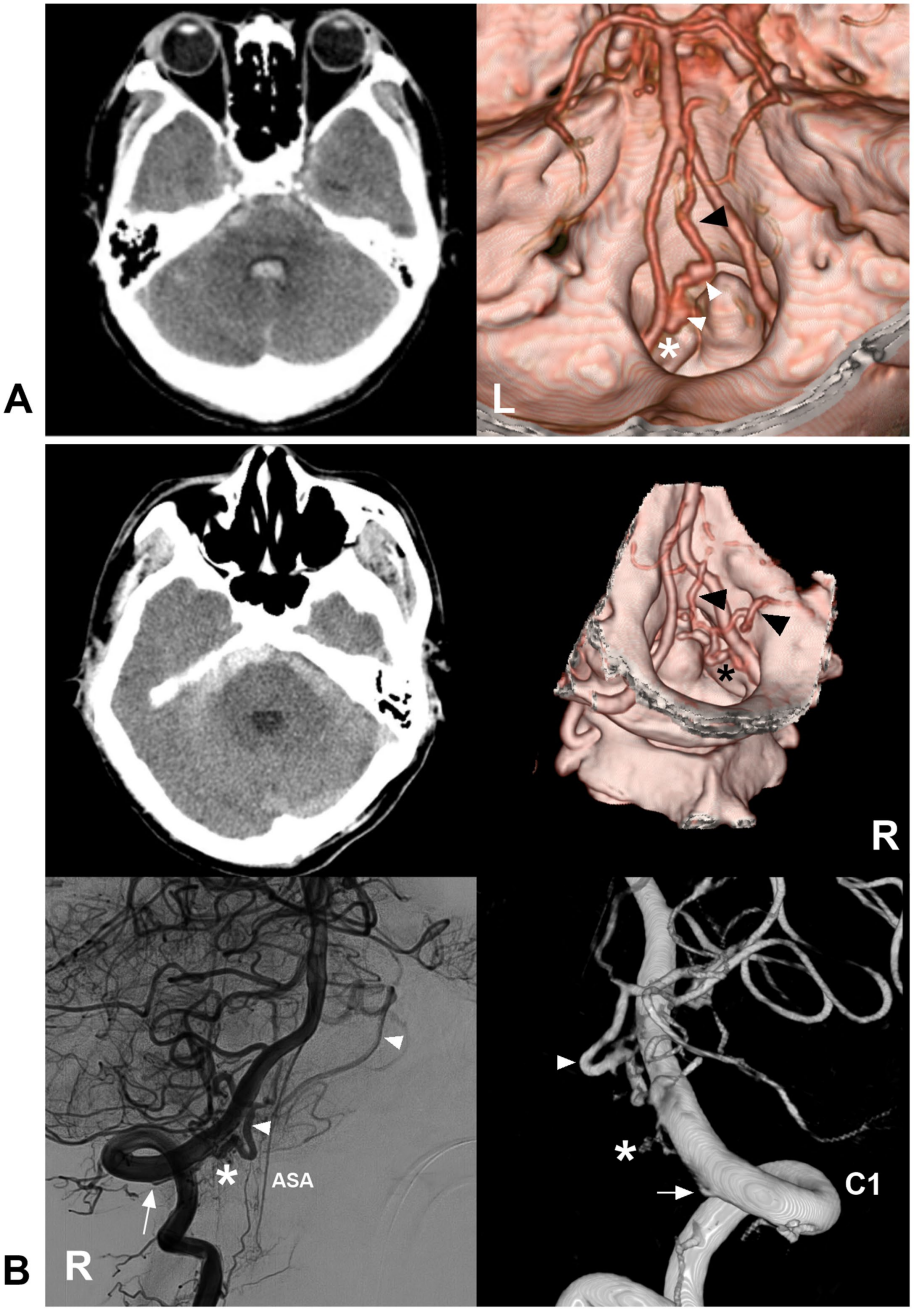
Ruptured CCJ AVF with upward drainage. **(A)** Left panel: CT showing subarachnoid hemorrhage and fourth ventricle hemorrhage; Right panel: CTA showing that a CCJ AVF (asterisk) had upward venous drainage (black arrowhead) with varices (white arrowheads). **(B)** Left upper panel: CT showing subarachnoid hemorrhage; right upper panel: CTA showing that a CCJ AVF (asterisk) had upward venous drainage (arrowheads); two-dimensional DSA (left below panel) and three-dimensional DSA (right below panel) showing a RAVF (asterisks) fed by the C1 radicular artery (arrows). The drainage went into the intracranial vein (arrowheads). AVF, arteriovenous fistula; C1, first cervical vertebrae; CCJ, craniocervical junction; CT, computed tomography; CTA, CT angiography; DSA, digital subtracted angiography; RAVF, radicular AVF.

**Figure 10:**
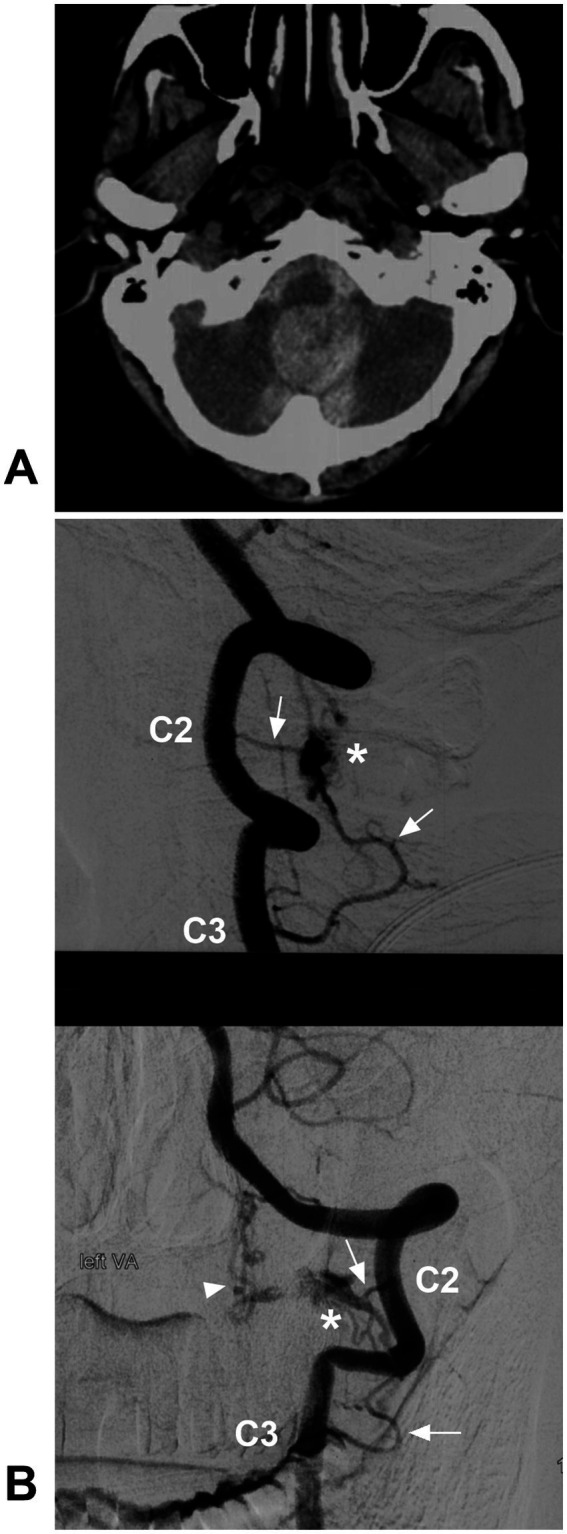
Ruptured CCJ RAVF with upward drainage. **(A)** CT showing subarachnoid and cisterna magna hemorrhage. **(B)** Lateral view (upper panel) and anterior posterior view (below panel) DSA of the VA showing an RAVF (asterisks) fed by the C2 and C3 radicular arteries (arrows) and with upward venous drainage (arrowhead in below panel). AVF, arteriovenous fistula; C2 and C3, second and third cervical vertebrae; CCJ, craniocervical junction; CT, computed tomography; DSA, digital subtracted angiography; RAVF, radicular AVF; VA, vertebral artery.

The drainage pattern of CCJ AVFs determines their clinical manifestation. CCJ AVFs with downward drainage mainly result in congestive myelopathy (Figure 8), whereas those with upward drainage usually result in hemorrhage due to draining vein rupture (Figures 9, 10) (4, 65, 66). In addition, increased hemodynamic stress may lead to varices of the draining veins in nearly 80% of high cervical CCJ AVFs (36, 53, 55).

Of these high cervical CCJ AVFs, DAVF tends to present with venous congestion; Intradural RAVF and PAVF tend to accompany aneurysm in fistula and varix on draining vein and may present with hemorrhage (2, 32, 33, 44, 67). In CCJ DAVFs, the unique gadolinium enhancement pattern presenting with a “missing-piece sign” on magnetic resonance imaging (MRI) has been reported as a unique radiologic finding to lead to the early diagnosis of CCJ DAVFs (68). The finding was defined as at least one focal geographic nonenhancing area within a long (>2 vertebral segments) and intensely enhanced segment (69).

### 4.2. Characteristics of each AVF

#### 4.2.1. DAVF

DAVFs are the most common AVFs in the high cervical CCJ region, accounting for 2.23% of brain and spinal DAVFs (4, 7, 33). They are defined as an abnormal shunt between a radiculomeningeal artery and a radicular vein along the dural sleeve of C1-C2 nerve roots and drain into the intradural veins (Figure 5A) (2, 36). Rarely, the external carotid artery can be involved as a feeder (33–36, 61, 70). High cervical CCJ DAVFs are often fed by dural arteries alone and attached to the dura, and the fistula is wide and irregular. CCJ DAVFs may have upward or downward intradural drainage or lateral epidural drainage (2, 33, 35, 71). For CCJ DAVFs, contrast-enhanced computed tomography angiography and magnetic resonance angiography were useful to detect vascular abnormalities, i.e., dilated draining veins (2).

ASA and PSA can be involved in CCJ DAVFs. According to the feeding arteries and their relationships with the ASA and PSA, Choi et al. (54) classified CCJ DAVFs into three types: type 1 was fed by the radiculomeningeal artery from the VA and was not associated with the ASA or PSA; type 2 was fed by the radiculomeningeal artery, and the radicular artery also supplied the PSA near the fistula point; type 3 had the characteristics of type 1 or type 2 DAVFs, except the ASA also contributed to the fistula; and type 3 was uncommon.

#### 4.2.2. RAVF

RAVFs are defined as the direct communication between a radicular (radiculomeningeal) artery and a radicular vein on spinal C1-C2 nerve roots (Figure 5B) (72, 73). They can be in the extradural or intradural space, and most of them have intradural drainage (3, 32, 33, 72). High cervical CCJ RAVFs have similar appearances to DAVFs, and they are often misdiagnosed as DAVFs (33). However, they are different. RAVFs are often fed by both dural and pial arteries and are located between the dura and spinal cord, usually supplied by the spinal pial artery, but involvement of these arteries is thought to be rare in DAVFs. (Figure 6) (32). RAVFs are often narrow and limited along the spinal nerve root (Figures 9B, 10) (32, 34, 59). Fusion imaging with DSA and MRI might reinforce the differential diagnosis for these two types (32).

#### 4.2.3. EAVF

High cervical CCJ EAVFs are not uncommon (33). They are usually in the ventral epidural space and are an arteriovenous shunt between the radicular and/or meningeal arteries and the epidural venous plexus without a nidus-like structure (Figure 5C) (2, 5, 74). When the epidural venous plexus is opacified directly by any arteries, it is considered an EAVF (32). According to the involvement of the spinal pial artery, they are divided into EAVF with/without pial feeders (5, 33). According to the involvement of the draining vein, EAVFs are divided into types A and B, of which type A fistulas have both epidural and intradural venous drainages, and type B fistulas have only the epidural draining vein (75).

High cervical CCJ EAVFs without the spinal pial artery as the feeder or intradural drainage are generally benign (76). Compared with other CCJ AVFs, EAVFs may have wider drainage, such as the suboccipital venous plexus or posterior cervical vein (32, 56).

#### 4.2.4. PAVF

High cervical CCJ PAVFs are characterized by direct arteriovenous communication without an intervening nidus on the spinal cord surface that consists of single or multiple holes and drains into a single vein (Figure 5D) (33, 77). Most CCJ PAVFs are located ventral or ventrolateral to the cervical cord (62). CCJ PAVFs may often have multiple feeding arteries from the radiculomedullary artery and/or spinal pial arteries and directly drain into the perimedullary veins (3, 33–35, 62). CCJ PAVFs tend to have a high flow nature, so they are often associated with venous varices in the fistulous site or draining vein.

## 5. Treatment options for high cervical CCJ AVFs

High cervical CCJ AVFs can often present as acute hemorrhage, myelopathy, brainstem dysfunction, radiculopathy, etc. (2, 4, 36, 44, 53, 61, 66, 78–81). For symptomatic or ruptured high cervical CCJ AVFs, treatment should be prompt, including open surgery, EVT or both (53). In general, EVT is useful for EAVFs, rather than other dural and intradural AVFs. Direct surgery may be safe for CCJ AVFs. Therefore, the optimal treatment modality should be examined in the anatomy and type of AVF. In asymptomatic CCJ AVFs without intradural drainage or pial feeder aneurysms, conservative treatment can be considered (32).

### 5.1. EVT

**Figure 11:**
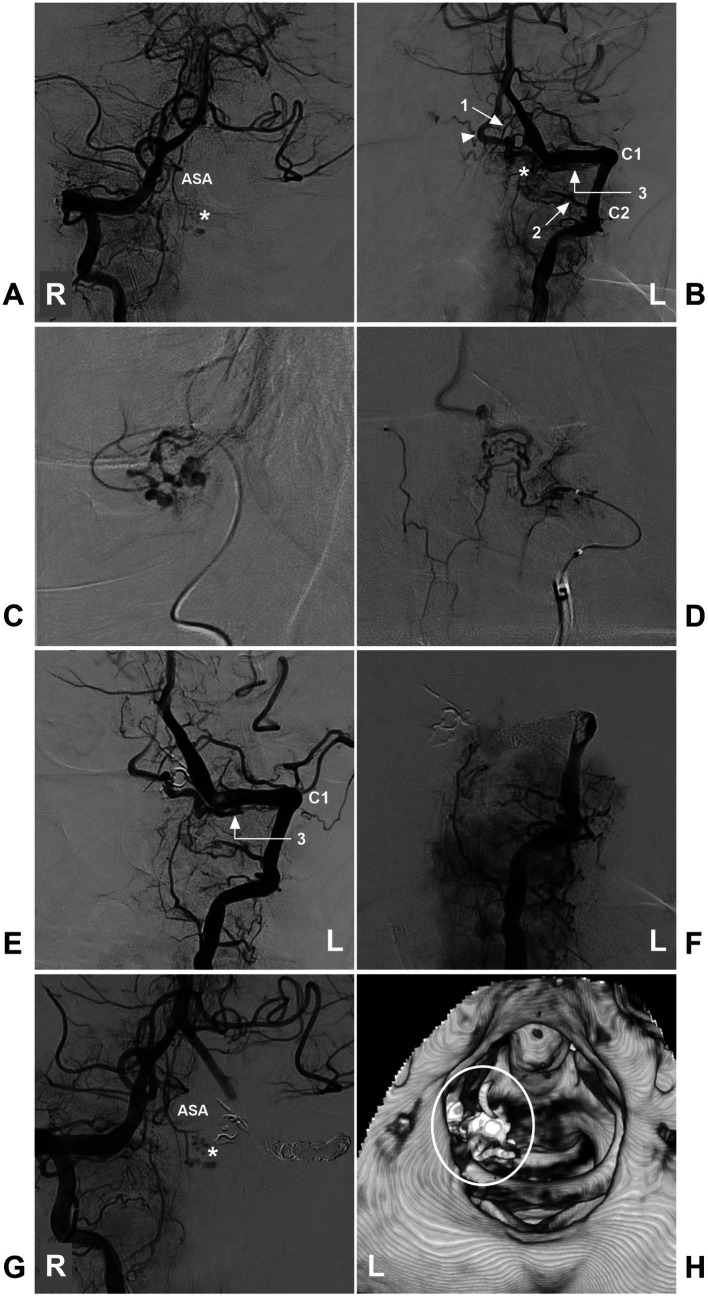
Incomplete EVT in a high cervical CCJ RAVF. **(A)** DSA of the left VA showing that the ASA supplied the AVF (asterisk). **(B)** DSA of the right VA showing that the AVF (asterisk) was fed by a brainstem perforating branch of the VA (arrow with number 1), the C2 (arrow with number 2) and C1 (arrow with number 3) radicular arteries, and the arrowhead indicates the draining vein. **(C)** Selective angiography of the brainstem perforating branch of the VA (arrow with number 1 in **B**) showing it to supply a part of the AVF. **(D)** Selective angiography of the C2 radicular artery (arrow with number 2) showing it to supply a part of the AVF. **(E)** After casting Onyx via two feeding arteries (numbers 1 and 2 in **B**), DSA of the left VA showing that the AVF was supplied by the C1 radicular artery (number 3 with arrow), confirming RAVF presentation. **(F)** DSA of the left VA showing that the VA was completely occluded by coiling, and the AVF could not be seen. **(G)** DSA of the right VA showing a part of the AVF (asterisk) was left. **(H)** Post-EVT CT reconstruction showing casting Onyx (ellipse), indicating the location of the RAVF. ASA, anterior spinal artery; AVF, arteriovenous fistula; C1-2, first and second cervical vertebrae; CCJ, craniocervical junction; CT, computed tomography; DSA, digital subtracted angiography; EVT, endovascular treatment; L, left; R, right; RAVF, radicular AVF; VA, vertebral artery.

**Figure 12:**
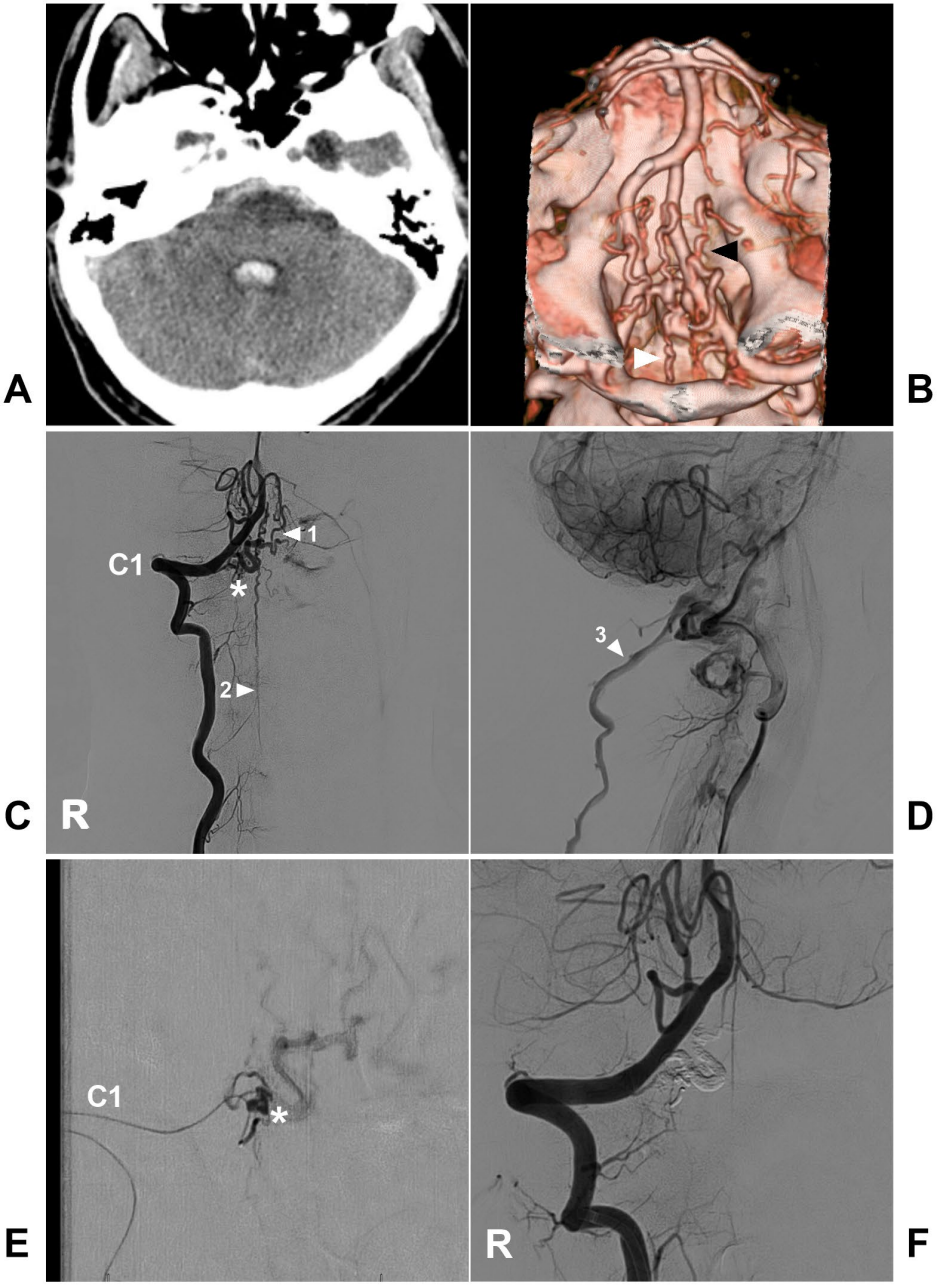
Complete EVT in a CCJ DAVF with a single feeding artery. **(A)** CT showing subarachnoid hemorrhage and fourth ventricle hemorrhage. **(B)** CTA showing multiple dilated abnormal veins (arrowheads). **(C)** Arterial phase DSA of the right VA showing a CCJ DAVF (asterisk) fed by the C1 radicular artery. The drainage went upward into the intracranial vein (arrowhead with number 1) and downward into the perimedullary vein (arrowhead with number 2). **(D)** Venous phase DSA of the lateral view showing drainage into the deep jugular vein (arrowhead with number 3). **(E)** Selective angiography of the microcatheter via the C1 radicular artery showing that the fistula was a DAVF (asterisk), and the microcatheter was obtained in the wedge position to perform Onyx casting. **(F)** Postoperative DSA showing that the DAVF was obliterated completely. C1, first cervical vertebrae; CCJ, craniocervical junction; CT, computed tomography; CTA, CT angiography; DAVF, dural arteriovenous fistula; DSA, digital subtracted angiography; EVT, endovascular treatment; R, right; VA, vertebral artery.

Although EVT reportedly has the benefit of being a minimally invasive procedure, it is not the best choice for high cervical CCJ AVFs (44, 80, 82). In EVT, the feeding arteries are usually very thin and tortuous and arise from the VA at a straight angle, which makes transarterial catheterization difficult for the fistula (66). Due to substantial feeder vessel tortuosity and diminutive caliber, transarterial EVT may harbor a risk of vessel perforation during microcatheterization. It is difficult to obliterate AVF without spinal cord infarction (Figure 11) (2, 83). EVT is only considered in CCJ AVFs with simple angioarchitecture, such as those with a single feeding artery and no pial feeding artery (Figure 12) (3). For type 1 CCJ DAVFs, EVT may be considered (54). For instance, in the report by Murase et al., a CCJ DAVF with a single feeding artery was successfully embolized by glue under the balloon-assisted technique (66).

In superselective cases of other CCJ AVFs, EVT can be attempted. For instance, in the report by Alshekhlee et al. (77), a CCJ PAVF was only fed by a thick and straight ASA, and it was successfully coiled via the ASA. Most EAVFs with intradural draining veins can be successfully treated by transarterial embolization with liquid embolic agents; therefore, EVT can be attempted in CCJ EAVFs. In addition, for CCJ EAVFs with accessible vein access, transvenous embolization can be attempted but remains challenging (84).

### 5.2. Open surgery

**Figure 13:**
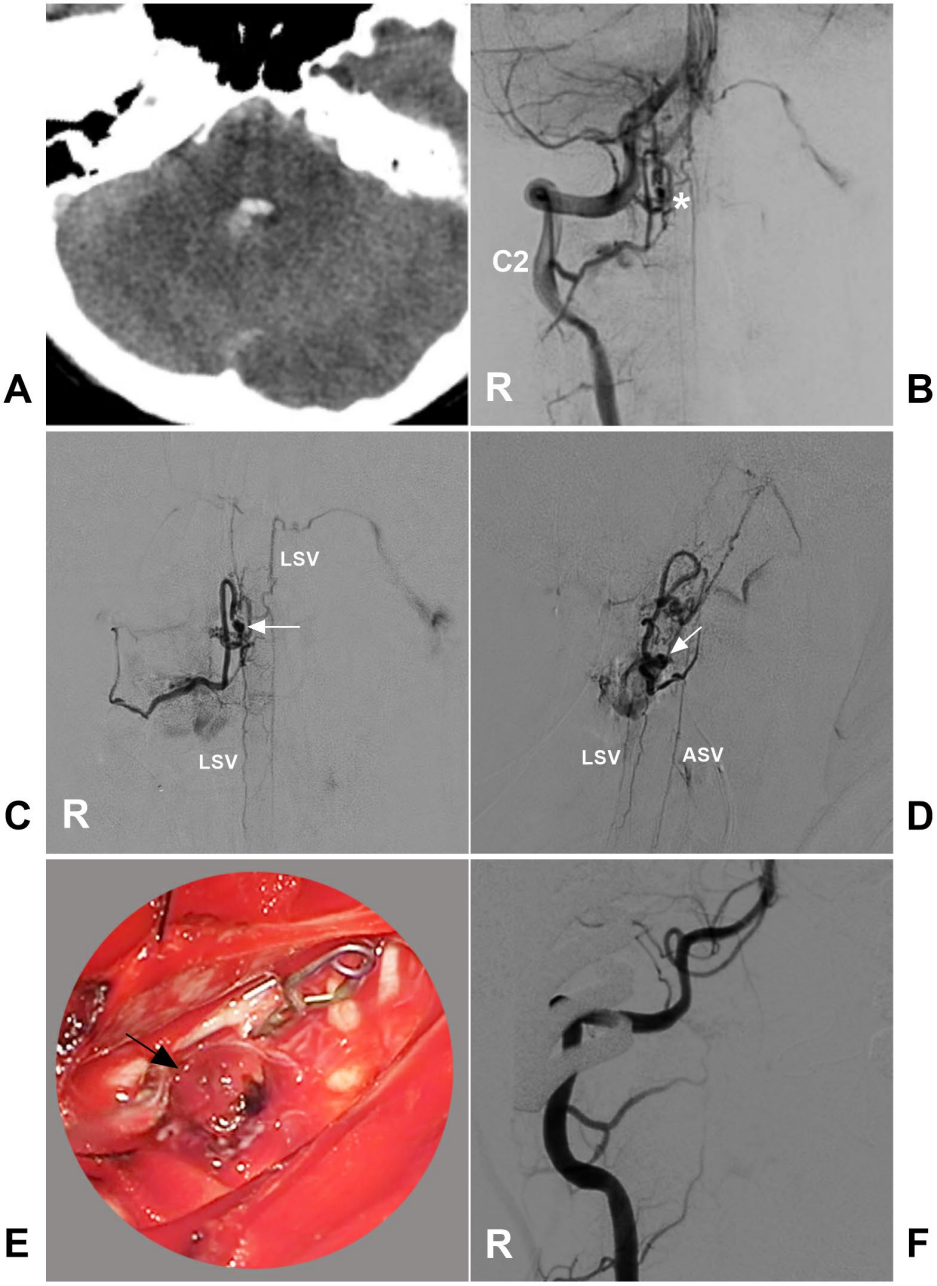
Open surgery in a CCJ PAVF. **(A)** CT showing subarachnoid hemorrhage and fourth ventricle hemorrhage. **(B)** Arterial phase DSA of the right VA showing a CCJ PAVF (asterisk) fed by the C2 radicular artery. **(C,D)** Late arterial phase DSA of the anterior posterior view **(C)** and lateral view **(D)** via a microcatheter showing the angioarchitecture of the PAVF. The feeding artery was the C2 radiculomedullary artery, there was aneurysm dilatation (arrows) in the fistula, and the draining veins included the upward and downward ASV and LSV. **(E)** Intraoperative image showing aneurysm dilatation (arrow) on the dorsal surface of the spinal cord, and the feeding artery was clipped. **(F)** Postoperative DSA showing that the PAVF was obliterated. ASV, anterior spinal vein; C2, second cervical vertebra; CCJ, craniocervical junction; CT, computed tomography; DSA, digital subtracted angiography; LSV, lateral spinal vein; PAVF, perimedullary arteriovenous fistula; R, right; VA, vertebral artery.

Compared to EVT, open surgery has a higher rate of obliteration and is still considered first-line management for high cervical CCJ AVFs (Figures 8, 13) (4, 5, 35, 56, 62, 66, 85, 86). For CCJ DAVFs and RAVFs with an intradural feeder, the surgical goal is to interrupt the intradural feeder and the draining vein by suboccipital craniotomy and C1 laminectomy. For CCJ DAVFs and RAVFs without an intradural feeder, the surgical goal is to interrupt the intradural draining vein (3, 56, 76). For PAVFs, the entire shunt system, including the proper feeders and drainers, was treated with coagulation (35).

During surgery for CCJ AVFs, it is crucial to distinguish the fistula, feeding arteries, draining vein, normal spinal artery, and normal spinal vein (35). Because it cannot reveal the location of an AVF in the operative field, microsurgery should be performed in a hybrid operating room with the assistance of intraoperative DSA; indocyanine green videoangiography is the minimum necessary requirement (34, 35, 56, 87). Using intraoperative DSA, the minimally invasive technique offers more precise but less destructive access than conventional far lateral suboccipital craniotomy (88). In addition, the fusion of rotation myelography and computed tomography is helpful to find the excised fistula structure (34, 72, 89). The assistance of the endoscope enables observation of the angioarchitecture of the AVF (62).

## 6. Prognosis and compilation

### 6.1. Prognosis

Good outcomes are defined by a modified Rankin scale (mRS) score of 0–2. In general, open surgery for high cervical CCJ AVFs can achieve good outcomes in more than 90% of cases (Table 1) (3, 4, 35, 36, 53, 90). In reports with large case series with more EVT attempts, the complete obliteration of CCJ AVF was low, and the prognosis of patients was poor, such as in the reports by Song et al. (7), Takai et al. (9), and Hiramatsu et al. (33) (Table 1). Good outcomes in EVT of CCJ AVFs are usually detailed in case reports, however, which do not accurately represent the universality of the effect of EVT in CCJ AVFs (61, 66, 77). In addition, EVT for CCJ AVFs has been associated with a higher incidence of recurrence (9, 34).

**Table 1:** Therapeutic outcomes of high cervical CCJ AVFs.

No.	References	Types of CCJ AVF	Treatment	Outcome
1	Kinouchi et al. (36)	Ten patients with 10 DAVFs	Open surgery to interrupt intradural draining vein in 10 DAVFs	All patients had good outcomes
2	Sato et al. (90)	Nine patients with 10 DAVFs and 9 PAVFs	Surgical interruption of intradural draining vein in 10 DAVFs; Coagulation and dissection of entire fistula structure in 9 PAVFs	All patients had good outcomes
3	Zhong et al. (4)	Thirty-eight patients with 40 DAVFs	Open surgery to interrupt the draining vein in 36 patients	91.7% patients had favorable outcomes
4	Kanemaru et al. (35)	Seven patients with 10 AVFs: 3 DAVFs, 1 PAVF, and 3 mixed DAVF and PAVFs	Open surgery to interrupt intradural draining vein in 6 DAVFs; Coagulated entire shunt system in 4 PAVFs	All patients had good outcomes
5	Goto et al. (3)	Twelve patients with 19 AVFs: 6 DAVFs, 5 RAVFs, 2 EAVFs, and 6 PAVFs	Open surgery in 8 patients: interrupting intradural feeding artery and daining vein in 5 patients; Only interrupting intraural draining vein in 3 patients	Overall outcomes were good
6	Takai et al. (9)	Ninety-seven patients with AVFs: 55 DAVFs, 17 RAVFs, 14 EDAVFs, and 11 multiple AVFs	Open surgery in 78 patients, EVT in 19 patients	Improved mRS scores were in 60% patients after open surgery and 37% patients after EVT
7	Takai et al. (33)	Fifty-four patients with 59 AVFs: 22 DAVFs, 17 RAVFs, 14 EAVFs, and 6 PAVFs	Open surgery in 28 AVFs, EVT in 15 AVFs, and both open surgery and EVT in 8 AVFs	Postoperative overall mRS scores improved. In DAVFs, the mRS scores improved significantly
8	Song et al. (53)	Twenty patients with 24 AVFs: 1 DAVF, 15 RAVFs, 2 EAVFs, and 6 PAVFs	Open surgery in 13 patients, EVT in 1 patient, and the combination of open surgery and EVT and in 6 patients	95% patients had favorable outcomes
9	Song et al. (7)	One hundred and thirteen patients with 122 AVFs: 87 DAVF, 15 RAVFs, 14 EAVFs, and 6 PAVFs	Open surgery in 98 AVFs, EVT in 7 AVFs, and both open surgery and EVT in 17 AVFs	81.4% patients had good outcomes
10	Choi et al. (54)	Sixteen patients with 16 AVFs	Open surgery in 4 AVFs, EVT in 2 AVFs, both open surgery and EVT in 10 AVFs	In 12 EVTs, 1 type 1 CCJ DAVF was completely cured without complications, 9 had residual lesions, and 2 had spinal cord infarction. After EVT, 10 AVFs were treated by open surgery

AVF, arteriovenous fistula; CCJ, craniocervical region; DAVF, dural AVF; EAVF, epidural AVF; EVT, endovascular treatment; mRS, modified Rankin Scale; PAVF, pial AVF; RAVF, radicular AVF.

Except for successful obliteration of CCJ AVFs by either open surgery or EVT, many factors can affect the prognosis of high cervical CCJ AVFs. In general, patients who present with hemorrhage may have better outcomes than those who present with venous congestion. Of these high cervical CCJ AVFs, DAVFs tend to present with venous congestion, and intradural RAVFs and PAVFs tend to present with hemorrhage; therefore, CCJ intradural and extradural AVF cases tend to have better outcomes than DAVF cases. In addition, younger age is predictive of a favorable outcome (10, 91). Certainly, prompt diagnosis also offers a greater chance of immediate obliteration and may optimize the outcomes (80).

### 6.2. Complication

For high cervical CCJ AVFs, complications were always accompanied by open surgery or EVT, including ischemic and hemorrhagic complications, hydrocephalus, and cerebrospinal fluid leakage (4, 7, 37, 92). In open surgery, the rate of complications is approximately 20%, especially in patients with massive hemorrhage or complex CCJ AVFs (4, 7, 8, 13, 93). When performing open surgery, a complete understanding of the angioarchitecture of CCJ AVFs was required to avoid injury to normal nerves and vessels.

Compared with open surgery, EVT has a higher rate of complications (Table 1). In the report by Takai et al. (9), the overall complication rates were 42% in the EVT group, and the ischemic complication rate for the EVT group was 26% for spinal and brainstem infarctions, which was more than threefold higher than that for the open surgery group. Therefore, EVT should be only cautiously performed in certain cases (35).

Given the risk of serious complications associated with the treatment of CCJ AVF, Inoue et al. (94) suggested that prophylactic intervention should be carefully considered in asymptomatic CCJ AVF. In addition, in their report, no case of asymptomatic CCJ AVF became symptomatic during the follow-up observation (94).

## 7. Summary

Rarely, AVFs occur in the CCJ region. Currently, it is accepted that CCJ AVFs should only be used to refer to AVFs at the C1-C2 levels. It is reasonable that the term ‘high cervical CCJ AVFs’ is more appropriate to use when discussing CCJ AVFs. High cervical CCJ AVFs can be divided into DAVFs, RAVFs, EAVFs and PAVFs. These four types of CCJ AVFs share similar clinical and imaging characteristics. When they become symptomatic, treatment is necessary. Most high cervical CCJ AVFs can be effectively treated with open surgery. EVT should only be used in certain AVFs with simple angioarchitecture. After appropriate treatment, a good prognosis can be obtained.

## Author contributions

JY contributed to the conception and design of the review. HS collection of data. JY and HS contributed to drafting the text and preparing the figures. All authors contributed to the article and approved the submitted version.

## Conflict of interest

The authors declare that the research was conducted in the absence of any commercial or financial relationships that could be construed as a potential conflict of interest.

## Publisher’s note

All claims expressed in this article are solely those of the authors and do not necessarily represent those of their affiliated organizations, or those of the publisher, the editors and the reviewers. Any product that may be evaluated in this article, or claim that may be made by its manufacturer, is not guaranteed or endorsed by the publisher.
